# Cost-effectiveness of tooth preservation versus implant for persistent periapical pathosis in mandibular second molars

**DOI:** 10.1186/s40729-026-00678-2

**Published:** 2026-04-06

**Authors:** Muhammad Muthar Shaikh, Nighat Naved, Asif R. Khowaja, Fahad Umer

**Affiliations:** 1 Department of Dentistry & Oral Health Sciences, Aga Khan University Hospital, Karachi, Pakistan; 2 Brock University, St. Catharines, ON Canada

**Keywords:** Cost-effectiveness analysis, Persistent apical periodontitis, Intentional replantation, Surgical endodontics, Dental implants

## Abstract

**Introduction:**

Persistent apical periodontitis in mandibular second molars presents a clinical decision between tooth preservation and extraction with implant replacement. Although intentional replantation (IR), surgical endodontic treatment, and implant therapy demonstrate acceptable outcomes, their long-term economic value remains unclear. This study evaluated the cost-effectiveness of tooth-preserving strategies versus extraction with implant placement.

**Materials and methods:**

A Markov model simulated 40-year-old patients with persistent apical periodontitis in a mandibular second molar from a United States (US) private payer perspective. Three strategies were compared: IR, surgical endodontic treatment, and extraction with implant placement. The model used 6-month half cycles over 39.3 years with 3% annual discount rate. Transition probabilities were derived from systematic reviews and clinical studies. Costs were obtained from the American Dental Association (ADA) 2022 survey adjusted to 2025 USD. Effectiveness was expressed as retained tooth-years or implant-years. Deterministic and probabilistic sensitivity analyses using 10,000 Monte Carlo simulations assessed uncertainty.

**Results:**

IR yielded 16.09 retained tooth-years at USD 2,676. Surgical endodontic treatment provided an additional 0.32 years corresponding to 16.41 tooth-years at an Incremental Cost-Effectiveness Ratio (ICER) of USD 4,279.66. Extraction with implant placement achieved 19.08 implant-years at an ICER of USD 580.60. Probabilistic analysis showed IR was cost-effective at lower willingness-to-pay thresholds, whereas implant placement became preferred beyond approximately USD 600.

**Conclusion:**

In mandibular second molars with persistent apical periodontitis, IR proved economically favorable in the short-term, while extraction with implant placement offered superior long-term effectiveness at reasonable incremental costs for patients with extended life expectancy.

**Graphical abstract:**

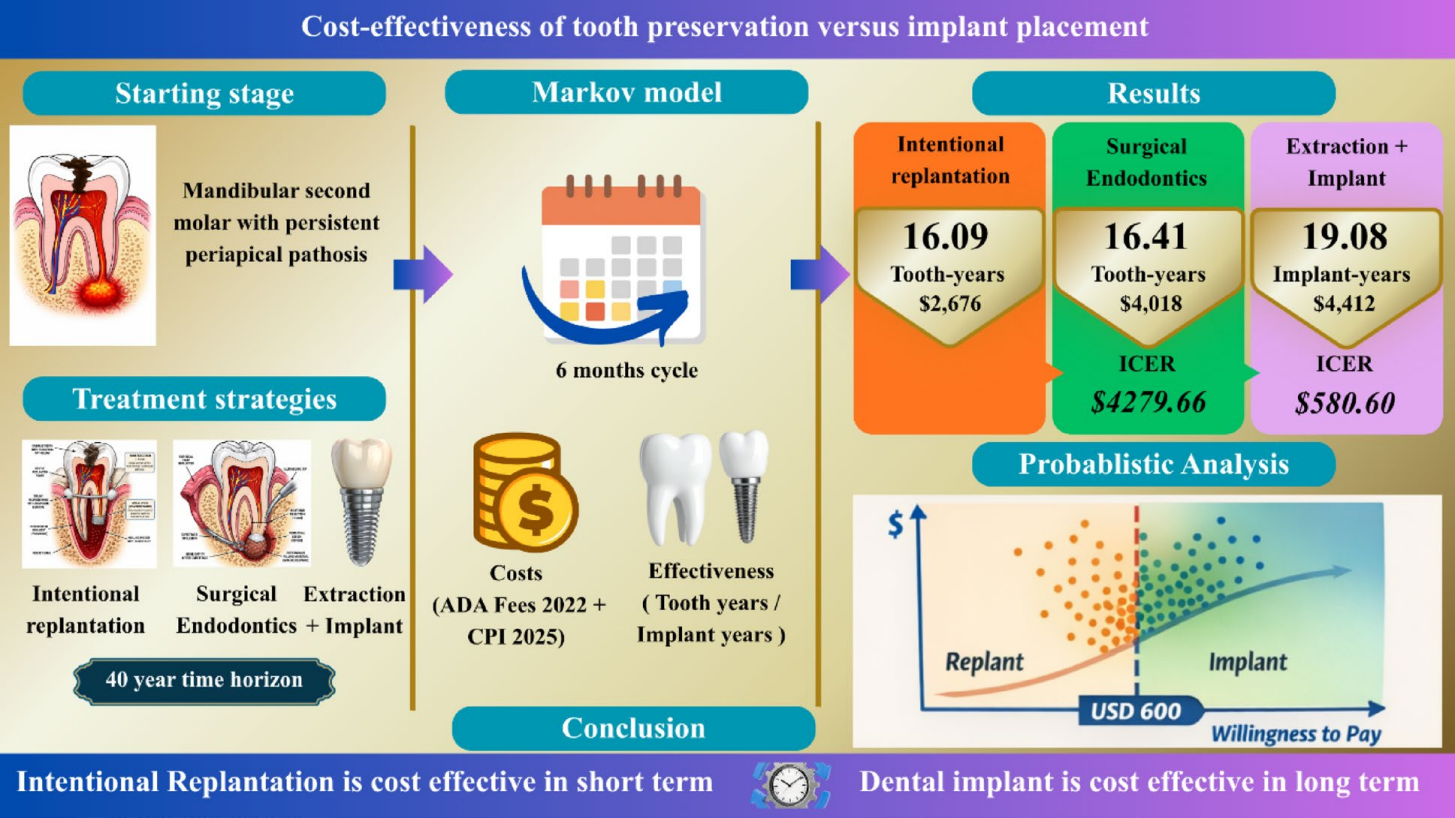

## Introduction

Persistent apical periodontitis despite adequate non-surgical endodontic intervention remains a common clinical problem in endodontic practice with prevalence of up to 40% [1]. This problem is particularly pronounced in permanent mandibular second molars which comprise a substantial proportion of posterior endodontic treatments, with failure rates of around 20% [2, 3]. These failures are attributed to anatomical constraints, including complex root canal anatomy, proximity to the inferior alveolar nerve, iatrogenic errors, limited surgical access, and restricted mouth opening in some patients compromising the predictability of further interventions [3, 4]. Selecting the appropriate management options for persistent apical periodontitis requires a robust understanding of distinct clinical and economic implications.

Intentional replantation (IR) is a conservative technique in which the tooth is atraumatically extracted, treated extra-orally with root-end resection and retrograde filling, and immediately replanted into its socket [5, 6]. Reported success rates for IR range from 70 to 90%, with outcomes highly dependent on the operator's expertise [7–9]. Surgical endodontic treatment has traditionally been regarded as the standard approach for managing persistent periapical pathology with success rates of 85–95% owing to advanced microsurgical techniques and biocompatible retrograde filling materials [10–12]. However, limited posterior access and proximity to the inferior alveolar nerve makes surgical endodontic treatment in mandibular second molars challenging. [13, 14]

While the aforementioned treatment options aim to preserve the patient’s natural teeth, some may advocate extraction to eliminate persistent periapical pathology followed by replacement with a dental implant [15]. Implant-supported restorations offer high long-term survival rates (> 95% at 10 years), predictable osseointegration, elimination of recurrent endodontic infection, and optimal load-bearing characteristics [16, 17]. However, implant placement requires substantial upfront financial investment, which may represent a significant barrier for many patients.

Although numerous studies have reported clinical success rates for intentional replantation, surgical endodontic treatment, and implant placement, the existing literature lacks comprehensive economic evidence to address the critical question of whether tooth preservation or extraction represents the optimal treatment strategy in managing persistent apical periodontitis [18–20]. Cost-effectiveness analyses are particularly valuable in such scenarios where multiple treatment modalities may demonstrate almost comparable clinical success rates but differ substantially in their economic implications. Therefore, the objective of this study was to estimate the incremental costs relative to outcomes comparing tooth preserving strategies (intentional replantation and surgical endodontic treatment) with extraction followed by implant placement for the management of permanent mandibular second molars with persistent apical periodontitis.

## Material and methods

### Study setting and population

This study adheres to the Consolidated Health Economic Evaluation Reporting Standards (CHEERS) 2022 [21]. A model-based cost-effectiveness analysis was conducted from a private payer perspective (out-of-pocket and/or third-party insurance) within the United States (US) healthcare system. We simulated a cohort of 40-year-old patients with a permanent mandibular second molar having persistent apical periodontitis following adequate non-surgical endodontic treatment. A Markov model was developed using TreeAge Pro Healthcare version 2025 (TreeAge Software, Inc., Williamstown, MA, USA), comparing the following three interventions:

1. Intentional replantation (IR)
2. Surgical endodontic treatment
3. Extraction followed by implant

The simulation extended until average life expectancy was reached (79.3 years), corresponding to a lifetime horizon of 39.3 years based on age-specific life expectancy estimates according to the National Vital Statistics Reports (Centre for Disease Control and Prevention, 2022) [22]. To maintain clarity and avoid statistical dependencies associated with multiple teeth per patient, the unit of analysis was tooth/implant in this study.

### Model description and assumptions

**Fig. 1:**
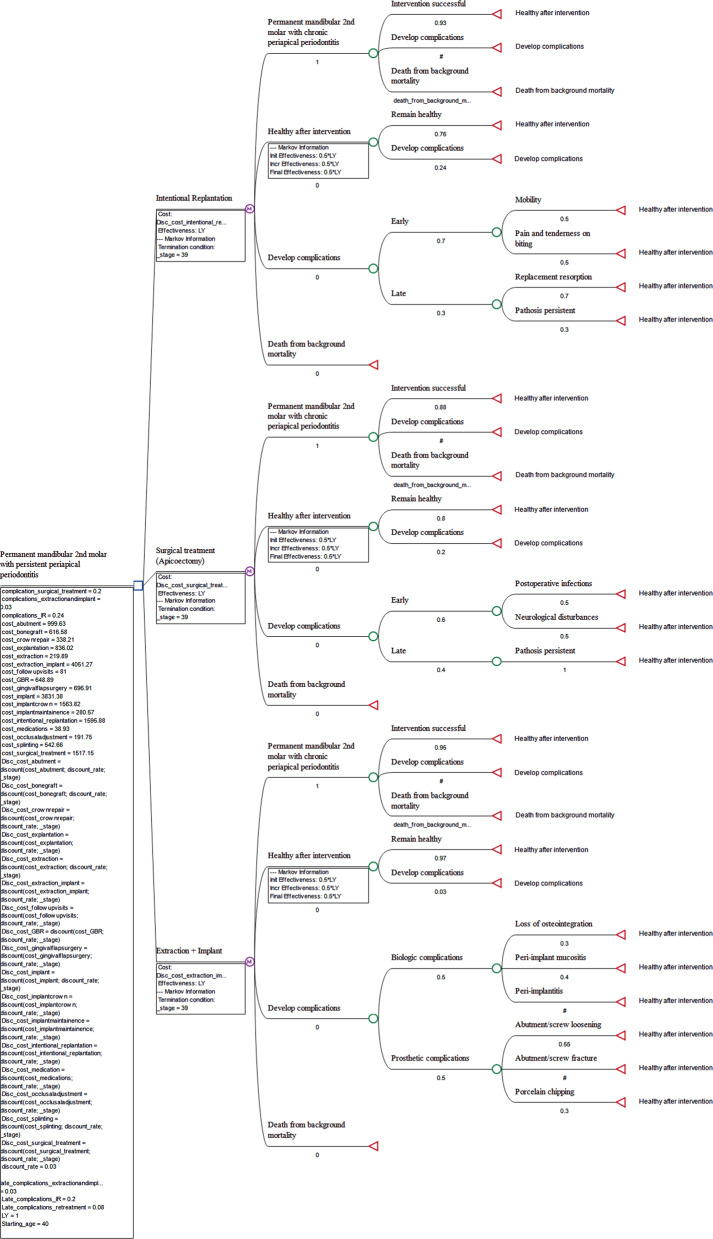
Markov model structure showing starting point (permanent mandibular second molar with persistent apical periodontitis following adequate non-surgical endodontic treatment.), health states, and transitions with probability values assigned. (# refers to 1 minus other probability within the same chance node)

A Markov state-transition model was developed to represent the clinical course of treatment, consisting of initial and subsequent health states. In each cycle, teeth or implants either remained in their respective state or transitioned to another health state based on predefined probabilities. Following assumptions were used to represent clinical/treatment pathways in the Markov model (Fig. 1):

1. All patients started in a disease state with a permanent mandibular second molar having persistent apical periodontitis following adequate non-surgical endodontic treatment.
2. Patients transitioned to a healthy functional state after receiving one of the three interventions: intentional replantation, surgical endodontic treatment, or extraction followed by implant.
3. Patients treated with any of the three strategies either remained in a healthy functional state or developed complications.
4. Complications were modeled as a separate health state subdivided into early or late complications in tooth-preserving treatments, or biological and prosthetic complications following implant placement.
5. In cases of failure following intentional replantation or surgical endodontic treatment, subsequent management included extraction of the affected tooth, followed by either implant placement or no further treatment.
6. Following failure of implant, subsequent treatment options included explantation with delayed implant placement or no further treatment.

Post intervention, both the teeth as well as implants either remained stable or transitioned between health states according to defined probabilities. Prosthetic rehabilitation was assumed to follow standard clinical protocols. The simulation was conducted in discrete 6-month cycles (half cycle correction). Model validation was performed through expert consultation with a health economist and internal calibration procedures that systematically varied key parameters to assess model consistency and logical coherence.

### Parameter estimation

A literature search was conducted in PubMed, Cochrane Library, EBSCO and Google Scholar to identify clinical studies reporting relevant outcomes. Procedural success and survival probabilities were extracted from systematic reviews, meta-analyses, and long-term clinical studies with follow-up periods of ≥ 5 years. Annual procedural failure rates were converted into 6-month transition probabilities under the assumption of constant hazard throughout the reported observation period.

### Outcome parameter

The primary health outcome was expressed as incremental cost per retained tooth-year/implant-year, consistent with previous economic evaluations in oral health [23–25]. Costs for all three interventions, encompassing surgical procedures, follow-up visits, prosthetic rehabilitation, and management of complications, were derived from the American Dental Association (ADA) Dental Fees Survey 2022 [26]. As no fee value for IR was reported in the 2022 ADA Dental Fees Survey, this cost was assumed based on expert elicitation from a U.S. based endodontist. Cost inputs were converted to 2025 U.S. Dollars using the U.S. Bureau of Labor Statistics Consumer Price Index for Dental Services (CPI-U). As complete CPI data for 2025 has not yet been published, the most recent available dental services CPI index (September 2025) was applied, resulting in a 4.7% inflation adjustment from 2022 to 2025 values [27]. The mortality and life expectancy data were obtained from the Center for Disease Control and Prevention, National Vital Statistics Reports [22].

The input parameters used to construct the model are presented in Table 1.

**Table 1:** Values for input parameters

Input parameters	Values
Intentional replantation *(D3470)*	1595.88 USD**
Extraction plus implant *(D7140)*	4051.27 USD*
Surgical endodontic treatment *(D3425 + D3426)*	1517.15 USD*
Abutment *(D6057)*	999.63 USD*
Implant supported crown *(D6066)*	1563.82 USD*
Crown repair *(D2980)*	338.21 USD*
Explantation *(D6100)*	836.02 USD*
Gingival flap surgery *(D4241)*	696.91 USD*
Bone grafting *(D7953)*	616.58 USD*
Implant maintenance procedures *(D6080)*	280.57 USD*
Medications *(D9630)*	38.93 USD*
Follow-up visits *(D9430)*	81 USD*
Guided bone regeneration *(D4266)*	648.89 USD*
Occlusal adjustment *(D9951)*	191.75 USD*
Splinting *(D4323)*	542.66 USD*
Starting age of the simulated cohort	40 years
Annual discount rate	3%
Intentional replantation (success probability)	0.93 [8]
Surgical endodontic treatment (success probability)	0.88 [28]
Extraction plus implant (success probability)	0.95 [16]
Complications following intentional replantation	0.24 [29]
Complications following surgical endodontic treatment	0.2 [10]
Complications following dental implants	0.03 [30]

*Costs of all modalities were simulated using ADA Dental Fees Survey 2022 + CPI (September 2025)

**Costs of IR confirmed by expert consultation with a U.S.-based endodontist

### Cost-effectiveness analysis

A Markov model was applied to determine the cost-effectiveness considering three clinical scenarios. The scenarios were evaluated based on their incremental cost-effectiveness ratios (ICERs) being calculated as the difference in costs divided by the difference in effectiveness between strategies. Following U.S. health economic evaluation standards, costs were discounted at an annual rate of 3% [31]. To assess internal consistency and robustness of the model, a one-way deterministic sensitivity analysis was performed by systematically varying critical parameters, including procedure costs and complication probabilities.

Probabilistic sensitivity analysis (PSA) was conducted using Monte Carlo simulation with 10,000 iterations, allowing all key input parameters to vary simultaneously. Transition probabilities were randomly sampled from the 5th to 95th percentiles of values documented in the published literature. Cost parameters were modeled using gamma distributions assuming a 10% variation around mean values to capture real-world cost variability. Complication probabilities were modeled using beta distributions with a conservative standard deviation of 0.02, consistent with hazard-based probability estimates [32, 33]. Finally, cost-effectiveness acceptability curves (CEACs) were generated to demonstrate the probability that each treatment strategy would be considered cost-effective across a range of willingness-to-pay (WTP) thresholds.

## Results

### Base case scenario

In the base-case scenario, a 40-year-old individual with persistent periapical periodontitis was followed for lifetime horizon, with a remaining life expectancy of 39.3 years. Three treatment strategies were compared: intentional replantation (IR), surgical endodontic treatment, and extraction followed by implant.

In the lifetime model, IR was associated with total cost of USD 2,676 yielding effectiveness of 16.09 years. This strategy therefore served as the reference comparator in incremental analysis. Surgical endodontic treatment yielded 16.41 years at a cost of USD 4,018, providing an incremental effectiveness gain of 0.32 tooth years (≈3.1 months) compared with IR but with an incremental cost of USD 1,342 corresponding to an ICER of USD 4,279.66 per tooth year gained. Extraction followed by implant provided effectiveness of 19.08 years, at a total cost of USD 4,412. Compared with IR, this strategy was associated with an incremental cost of USD 1,736 and the highest incremental effectiveness gain of 2.99 retention years (≈3 years), yielding an ICER of USD 580.60 per implant year gained (Table 2).

**Table 2:** Cost-effectiveness ranking report; intentional replantation and extraction followed by implant over lifetime. Extraction followed by implant placement was associated with an additional health benefit of 2.99 implant-years (≈ 3 years) at the expense of increased overall cost (USD 1736), resulting in an incremental cost-effectiveness ratio (ICER) of USD 580.60 per retained implant-year

Interventions	Cost (USD)	Incremental cost	Retained tooth/implant years (TY/IYs)	Incremental retained TY/IYs	ICER (Incremental cost/Incremental TY/IYs) USD/TY/IYs
Intentional replantation (IR)	2,676	–	16.09	–	
Surgical endodontic treatment	4,018	1,342	16.41	0.32	4,279.66
Extraction + Implant	4,412	1,736	19.08	2.99	580.60

*TY* Tooth years, *IY* Implant years, *ICER* incremental cost-effectiveness ratio, *USD* United States Dollar

#### Sensitivity analysis:

**Fig. 2:**
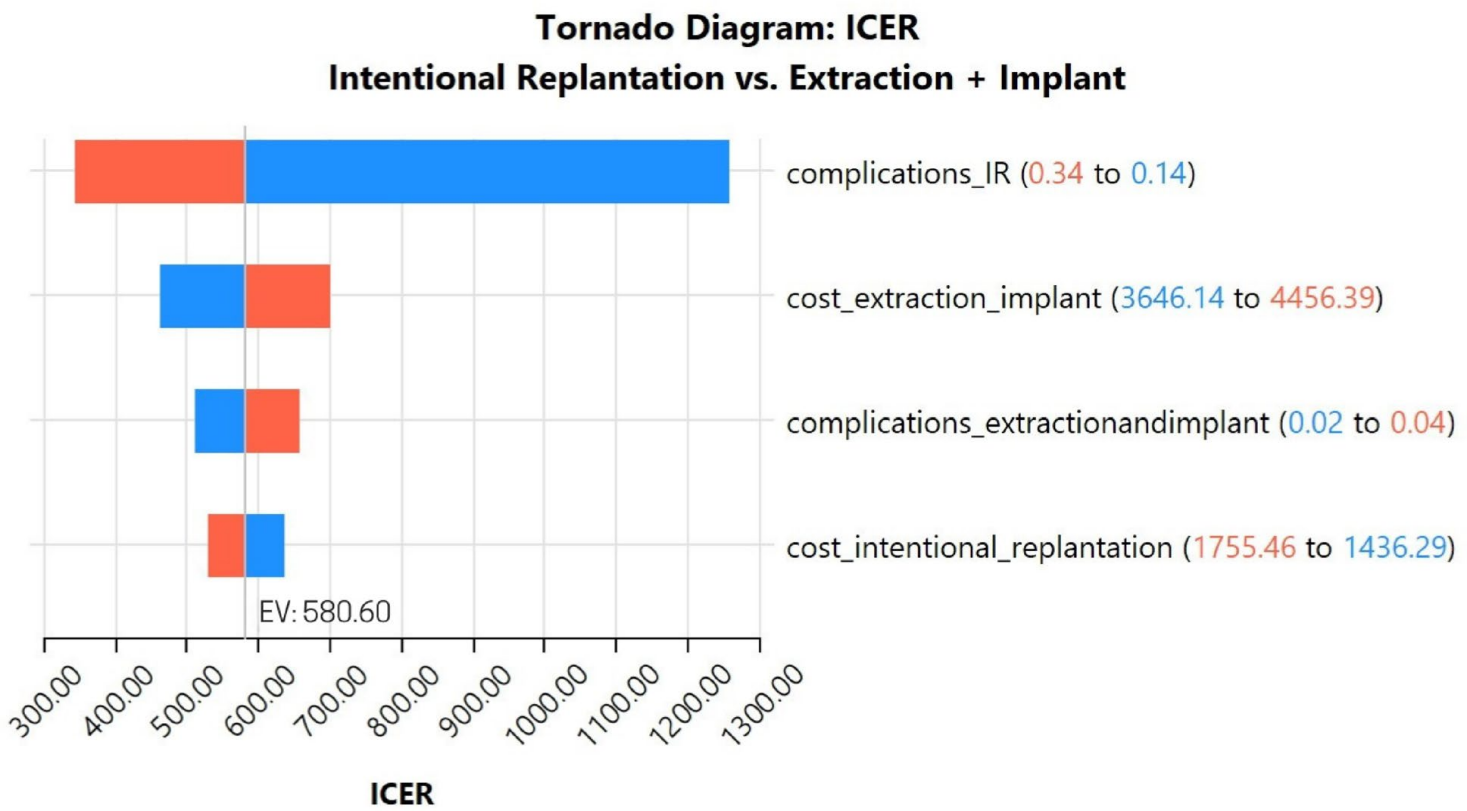
Tornado diagram for sensitivity analysis. Base case ICER was USD 580.60 per implant LY gained. Note the ICER variations from slight changes in input parameters, with blue bars indicating a decrease when parameter values were reduced and red bars showing an increase when the values were inflated

A deterministic sensitivity analysis as depicted in Fig. 2 revealed that ICER was most sensitive to complications linked to IR, followed by costs of extraction and implant placement. Changes in the probabilities of complications associated with extraction and implant placement, as well as costs of IR, had a lesser impact on the model.

### Probabilistic sensitivity analysis

Probabilistic sensitivity analysis was employed using Monte Carlo simulation with 10,000 iterations to estimate mean ICERs within 95% confidence intervals.

**Fig. 3:**
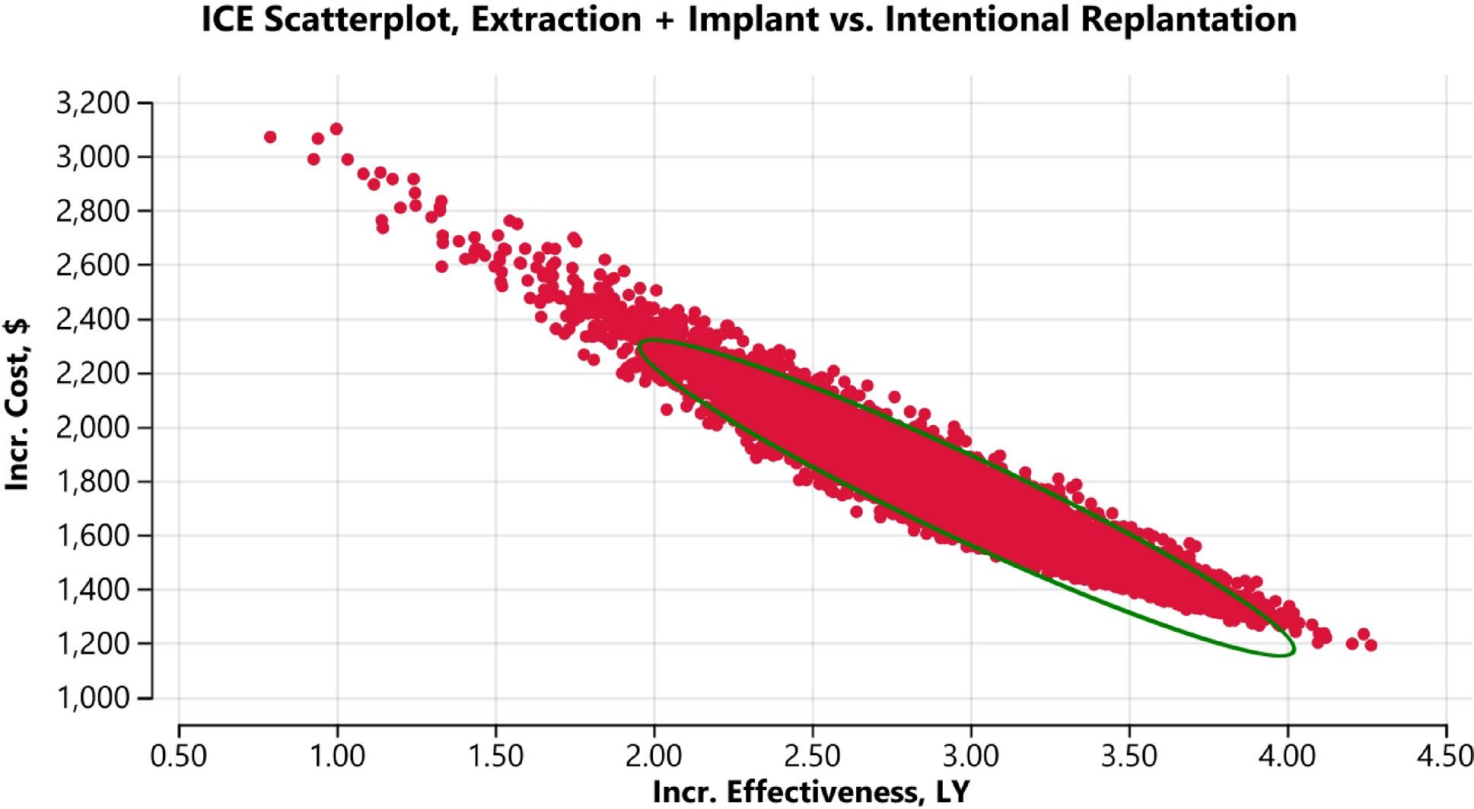
Cost-effectiveness plane. The ellipse represents the ICERs within 95% credible Intervals. Horizontal and vertical axes show the effectiveness and cost differences of intentional replantation and extraction followed by implant placement. The red dots represent scenarios in which ICERs were in the North-East quadrant representing increased cost and increased effectiveness of implants

In the first scenario when IR was compared with extraction and implant, (Fig. 3), the latter strategy demonstrated positive incremental effectiveness relative to the former. For most iterations, ICERs were distributed in the North-East quadrant of the cost-effectiveness plane, indicating higher costs and greater effectiveness for extraction with implant placement compared with IR.

**Fig. 4:**
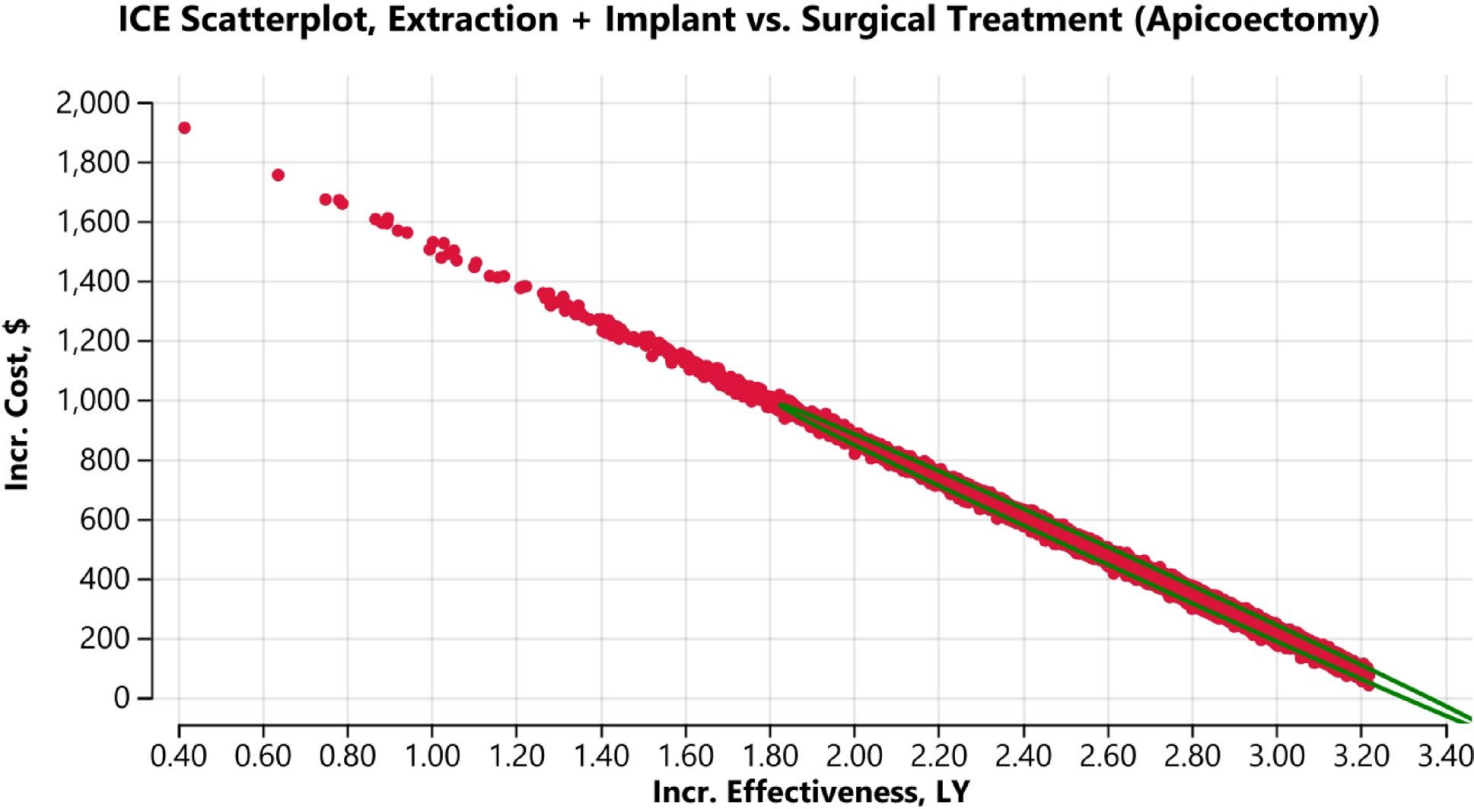
Cost-effectiveness plane. The ellipse represents the ICERs within 95% credible Intervals. Horizontal and vertical axes show the effectiveness and cost differences of surgical endodontic treatment and extraction followed by implant placement. The red dots represent scenarios in which ICERs were in the North-East quadrant representing increased cost and increased effectiveness of implants

Similarly, in the second scenario, when surgical endodontic treatment was compared against extraction and implant (Fig. 4), the majority of ICERs were again located in the North-East quadrant, reflecting a comparable trade-off between increased cost and improved effectiveness.

**Fig. 5:**
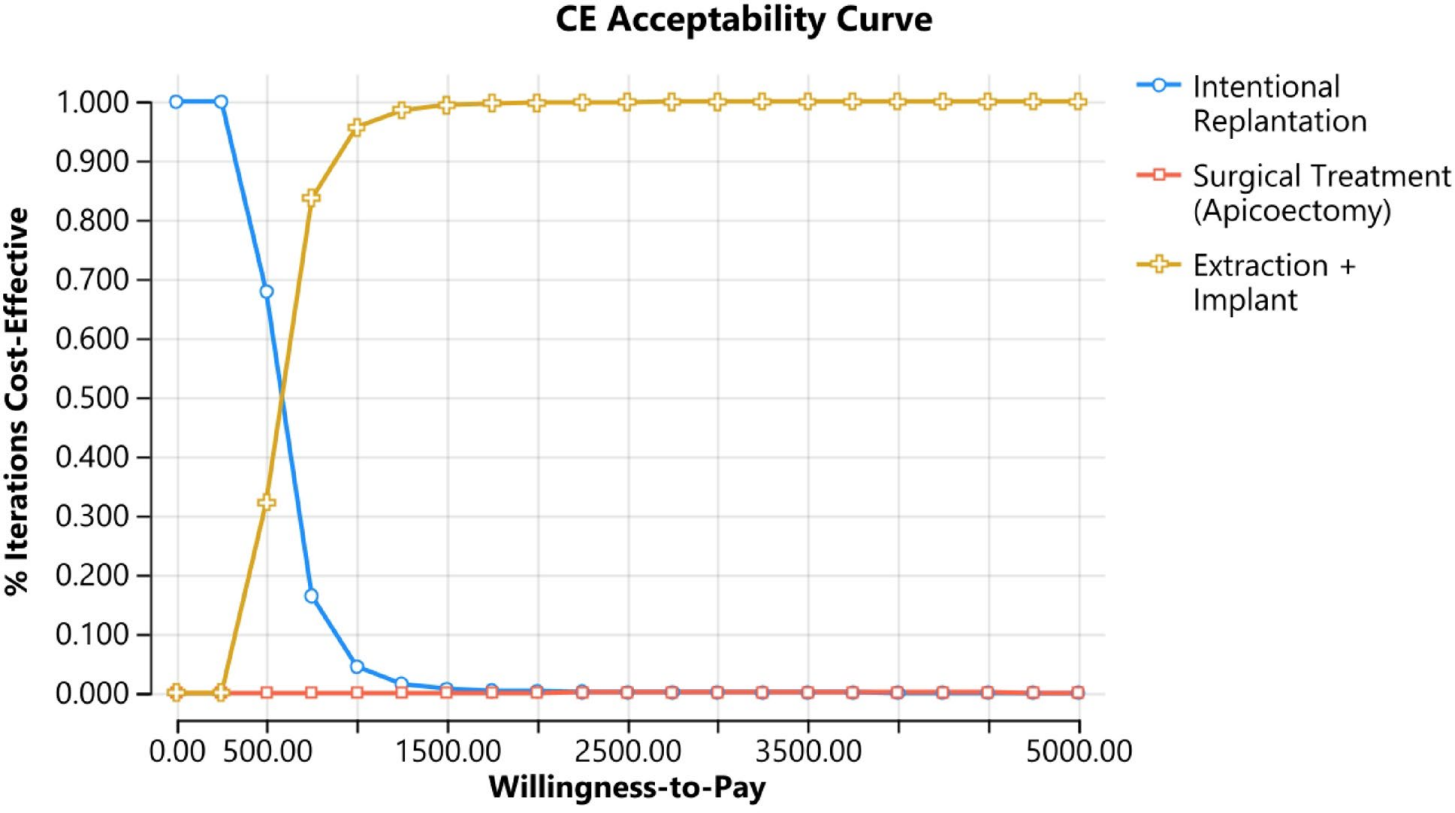
Cost-effectiveness acceptability curves; IR was cost-effective at lower WTP values (100.0% acceptable below 500 USD) whereas by increasing the values of WTP threshold extraction followed by implants were a cost-effective treatment option (80% acceptable at 750 USD)

To determine the cost effectiveness of individual strategies against a range of WTP thresholds, a cost-effectiveness acceptability curve (CEAC) was plotted (Fig. 5). At low WTP threshold (< 500 USD) IR exhibited a 100% probability of being cost-effective, reflecting its position as the least expensive option. However, as WTP increased beyond USD 500, the probability of intentional replantation being cost-effective declined progressively. Extraction followed by implant placement demonstrated a rapid increase in cost-effectiveness probability as WTP thresholds increased. At a WTP of approximately USD 750, extraction followed by implant placement became the preferred strategy, achieving a probability exceeding 80% whereas surgical endodontic treatment exhibited minimal probability of being cost-effective across the entire range of WTP values evaluated.

## Discussion

This study addresses an important clinical quest as to whether save a tooth or extract it. Using a Markov model-based cost-effectiveness analysis, this study evaluated the economic and effectiveness trade-offs between these competing strategies in managing persistent periapical pathology in mandibular second molars. The findings indicated that although tooth-preserving strategies were associated with lower initial costs, extraction followed by replacement with implant provided greater long-term effectiveness hence emerging as the most cost-effective strategy at commonly accepted WTP thresholds.

In the analysis, the economic performance of tooth preservation was primarily driven by two interrelated factors: the long-term durability of preserved tooth, surviving complications and maintaining function over time; and eventual transition to extraction followed by implant placement in cases of failure. Within this spectrum, IR represented the most conservative approach and was associated with the lowest initial cost. Although IR was associated with short-term cost advantage, its long-term effectiveness may have been limited by biological complications, increasing the probability of failure [29]. As failures accumulated over time, the need for additional interventions substantially increased cumulative costs. Consequently, attempts to save the tooth became not only more costly than immediate replacement with an implant but also consistently less effective over a lifetime horizon.

This finding is consistent with prior economic analyses indicating that IR may demonstrate favorable cost-effectiveness in the short to intermediate term due to lower upfront costs, however, its cost-effectiveness decreases when longer follow-up periods and repeated failures are taken into account [20]. In line with this, contemporary clinical evidence suggests that IR can achieve favorable survival rates often exceeding 80% at mid-term follow-up when strict case selection is ensured; however, this does not necessarily translate into superior long-term economic value; so, it is important to distinguish between a treatment’s biological feasibility and its overall lifetime cost-effectiveness. [34]

Surgical endodontic treatment demonstrated marginally greater effectiveness than IR but at a substantially higher incremental cost, resulting in an unfavorable cost-effectiveness profile. In the base-case analysis and across all probabilistic simulations, surgical endodontic treatment was consistently dominated by extraction followed by implant placement. Although modern endodontic microsurgery has demonstrated high success rates in controlled clinical environments, the present model focused specifically on mandibular second molars, where anatomical constraints such as thick cortical bone, limited access, lingual root inclination, and proximity to the inferior alveolar nerve may increase technical difficulty, complication risk, and overall treatment costs. Consequently, in mandibular second molars with persistent apical pathology, surgical endodontic treatment may represent a biologically reasonable option in selected cases but performs poorly as an economically efficient strategy when long-term outcomes and downstream interventions are considered.

In contrast, extraction followed by implant placement yielded the highest long-term effectiveness and emerged as the preferred strategy across a wide range of WTP thresholds. Although associated with higher upfront costs, implant therapy demonstrated durable outcomes with a lower probability of reintervention over time. These characteristics translated into superior retained functional years and favorable incremental cost-effectiveness ratios. This finding also aligns with the previous economic models, where implant placement was associated with predictable long-term survival and relatively stable maintenance requirements, advantages that become increasingly pronounced as patient’s life expectancy increases [20]. From a long-term perspective, replacing the tooth with an implant rather than repeatedly attempting to preserve it may represent a more predictable and economically efficient strategy in mandibular second molars with persistent apical pathology.

In economic analyses, WTP threshold represents a central determinant of decision-making, because it defines the maximum value that society or decision-makers are prepared to accept for a given health benefit. Accordingly, whether a tooth-preserving or tooth-replacing strategy is favored depends not only on clinical effectiveness but also on how incremental costs and outcomes align with the chosen WTP threshold. [35]

Consistent with these principles, the results of the present analysis demonstrated that the preferred strategy varied according to the assumed WTP threshold. At lower WTP levels, IR emerged as the most cost-effective tooth-preserving option. In contrast, surgical endodontic treatment was not cost-effective at any WTP threshold. As the WTP threshold increased, extraction followed by implant placement became increasingly favored. Specifically, implant replacement became cost-effective once the WTP threshold exceeded approximately USD 600, consistent with the observed ICER of USD 580.60 per additional retained implant year and achieved near-universal cost-effectiveness at higher thresholds (e.g., USD 1,000). Notably, this value is substantially lower than the WTP values for single-tooth implant replacement reported in the literature at USD 2,600 [36]. Conversely, WTP thresholds for tooth preservation strategies have not been established in the literature, limiting direct cost-effectiveness comparisons across preservation modalities.

Several limitations of the study should be acknowledged. The transition probabilities and complication rates were derived from heterogeneous sources with variable follow-up durations, clinical settings, and operator expertise, which may not fully reflect real-world outcomes. IR and surgical endodontic treatment are highly operator-dependent procedures, which can substantially influence success rates; however, the model applied fixed transition probabilities and did not account for this variability. Moreover, the analysis was conducted from a private payer perspective and in the context of US healthcare, thus may have restricted generalizability to other healthcare systems. The effectiveness outcome was expressed as retained tooth-years or implant-years, reflecting treatment longevity but not patient-centered outcomes such as quality of life, functional satisfaction, pain, or patient preferences. Future analyses incorporating these values would provide a more comprehensive assessment of patient’s perspective.

Additionally, strategies like non-surgical endodontic retreatment, delayed implant placement, prosthetic options other than dental implant, and no treatment after extraction were not modeled separately. Notably, extraction without replacement represents a clinically relevant and cost-saving alternative, particularly in patients with limited financial resources, systemic comorbidities precluding implant surgery, or insufficient bone volume. Although this option was incorporated in the model as a potential post-failure pathway, it was not evaluated as a primary strategy. In the context of mandibular second molars, this may be functionally tolerated in some patients, albeit with long-term risks of supra-eruption of the opposing tooth, mesial drift, and altered occlusal loading. Future models should incorporate these pathways to better reflect the complexity of clinical decision-making.

Finally, the present “save or extract” framework was intentionally limited to permanent mandibular second molars, a tooth group characterized by unique anatomical constraints, restricted surgical access, and increased technical complexity. Consequently, the findings should not be extrapolated to other tooth types or regions, such as anterior teeth, premolars, or maxillary molars, where anatomical considerations, surgical predictability, and long-term outcomes may differ substantially.

Future research should extend similar cost-effectiveness analyses to other tooth types and anatomical regions to determine whether the economic trade-offs between tooth preservation and implant replacement observed in this study are consistent across the dentition.

## Conclusion

Findings from this study suggest that in mandibular second molars with persistent apical periodontitis, IR represents an economically favorable strategy in short term whereas extraction with implant placement offers superior long-term effectiveness at reasonable incremental costs for patients with extended life expectancy. However, these results should be interpreted with caution, as individual patient factors, clinical circumstances, and preferences can influence treatment selection.

## Data Availability

The data that support the findings of this study are available on request from the corresponding author.
